# Fucoxanthin Attenuates Oxidative Damage by Activating the Sirt1/Nrf2/HO-1 Signaling Pathway to Protect the Kidney from Ischemia-Reperfusion Injury

**DOI:** 10.1155/2022/7444430

**Published:** 2022-01-28

**Authors:** Hu Mao, Lei Wang, Yufeng Xiong, Guanjun Jiang, Xiuheng Liu

**Affiliations:** ^1^Department of Urology, Renmin Hospital of Wuhan University, Wuhan, 430060 Hubei, China; ^2^Department of Urology, The Fifth Hospital of Wuhan, Wuhan, 430050 Hubei, China

## Abstract

Oxidative stress is a key component of renal ischemia/reperfusion (I/R) injury. Fucoxanthin (Fx), a marine carotenoid with enhanced antioxidant capacity, acts as a ROS inhibitor in diseases such as ischemic stroke and acute lung injury. We hypothesized that fucoxanthin could attenuate renal I/R-induced oxidative damage. C57BL/6 mice (*n* = 30) were randomly assigned to sham, IR, IR + DMSO, and IR + Fx (25, 50, and 100 mg/kg) groups. The renal I/R injury was induced by clamping the left kidney nephron tip in mice. Fucoxanthin was injected intraperitoneally 24 hours before surgery. Compared with the IR group, pretreatment with fucoxanthin significantly improved renal dysfunction and tissue structural damage and inhibited ROS levels and apoptosis. Consistent results were observed in HK-2 cells. Besides, we found that renal I/R resulted in decreased expression of Sirt1, Nrf2, and HO-1, while fucoxanthin upregulated the expression of Sirt1, Nrf2, and HO-1. The protective effects of fucoxanthin were significantly reversed by EX527 (a selective inhibitor of Sirt1) or si-Sirt1. In conclusion, our study investigated the protective effect of fucoxanthin against renal I/R injury, and the underlying mechanism may be related to the activation of the Sirt1/Nrf2/HO-1 signaling pathway by fucoxanthin to attenuate oxidative stress-induced apoptosis.

## 1. Introduction

Acute kidney injury (AKI) is a common clinical syndrome, usually caused by renal surgery and sepsis, characterized by a sudden decrease in glomerular filtration rate during the initial phase [1]. AKI is associated with adverse clinical outcomes, including chronic kidney disease [2] and increased mortality [3]. Significant progress has been made in recent years in the emergency treatment of AKI, but the incidence and mortality of AKI remain high, and therefore, effective therapeutic agents are still lacking [4, 5]. Renal ischemia-reperfusion (I/R) injury is widely recognized as one of the most common clinical causes of AKI [6]. I/R usually causes significant renal tubular injury. Studies have shown that elevated levels of reactive oxygen species (ROS) in the affected tissues are an important cause of tubular epithelial cell death and renal injury after renal I/R [7]. During ischemia, mitochondria are severely damaged in tissues, and the function of complexes I and III, which maintain mitochondrial function, is altered, leading to ROS production [8, 9]. During reperfusion, reactivation of aerobic metabolism and hyperoxia also alters the function of mitochondrial complexes, leading to increased production of ROS. ROS's excessive production exceeds the cellular antioxidant capacity, further inducing tissue damage and apoptosis [10]. With the increased morbidity and mortality of renal I/R injury, there is an urgent need to find new therapeutic targets and develop new treatment approaches.

Silent information regulator 1 (Sirt1) is an NAD^+^- (nicotinamide adenosine dinucleotide-) dependent deacetylase that can regulate a variety of cellular activities including gene transcription, senescence, cellular stress responses, glucose metabolism, and energy homeostasis through deacetylation of diverse factors [11–13]. Sirt1 can attenuate oxidative stress, inflammation, and apoptosis as well as regulating mitochondrial function and autophagy [14–18]. Several studies have shown that Sirt1 has protective effects against I/R-induced liver, heart, and brain injury [19–21]. In addition, the renoprotective effects of Sirt1 have been demonstrated in different renal diseases [22, 23]. For example, Sirt1 attenuates renal I/R injury by reducing apoptosis and promoting cell regeneration [24].

Nuclear factor erythroid 2-related factor 2 (Nrf2) is a major transcriptional regulator of intracellular antioxidant proteins. Nrf2 reduces ROS levels by regulating the expression of downstream antioxidant genes HO-1 and NQO1 to prevent oxidative stress-induced tissue damage and apoptosis [25, 26]. Numerous studies have shown that the level of intracellular ROS levels after renal I/R is closely related to the degree of renal injury [27, 28]. In addition, several studies have confirmed that Sirt1 can contribute to Nrf2 nuclear translocation, enhance the DNA binding activity and transcriptional activity of Nrf2, and upregulate HO-1 expression [29–31]. Therefore, upregulation of Sirt1 activation of Nrf2 to suppress oxidative stress represents a potential strategy to improve the treatment of renal I/R injury.

Carotenoids are widely found in nature and are natural pigments that play an important physiological function. Hundreds of marine carotenoids have been discovered, and reports on the biological functions of marine carotenoids have been gradually increasing in recent decades [32]. Fucoxanthin (Fx) is one of the most abundant marine carotenoids found mainly in brown seaweeds, accounting for about 10% of the total natural carotenoid production [33]. Fucoxanthin exerts biological activity in a variety of diseases, including obesity, cancers, and diabetes [34–36]. In terms of antioxidant capacity, fucoxanthin contains a unique chemical structure (Supplementary Figure S1) including a propadiene bond and 5,6-monocyclic oxides with an enhanced antioxidant capacity [37]. Published studies have shown that fucoxanthin has unique advantages in the treatment of several oxidative stress-related diseases, such as ischemic stroke, subarachnoid hemorrhage, acute lung injury, and atherosclerotic cardiovascular disease [38–41]. In addition, as an edible carotenoid, fucoxanthin has a good safety profile. When the daily intake of fucoxanthin reaches 200 mg/kg, there is still no apparent teratogenic effect in vivo, and it is considered a very safe antioxidant [42]. However, there are no reports on whether fucoxanthin is beneficial to renal I/R injury.

In this study, we investigated whether fucoxanthin could prevent renal I/R injury and explored the potential mechanism. We established a mice renal I/R injury model and a human renal proximal tubular epithelial cell line (HK-2) hypoxia/reoxygenation (H/R) injury model. The mice and HK-2 cells were pretreated with fucoxanthin, and changes in renal function, pathological injury, oxidative levels, and apoptosis were detected. Deeply, we explored whether the protective effect of fucoxanthin was related to the activation of the Sirt1/Nrf2/HO-1 signaling pathway and further validated the specific mechanism by inhibiting Sirt1.

## 2. Materials and Methods

### 2.1. Experimental Animals and Renal I/R Model

Adult male C57BL/6 mice (age, 7-8 weeks; weight, 23-25 g) were provided by the Wuhan University (Wuhan, China) Experimental Animal Center. Mice were housed in a specific pathogen-free facility (temperature, 21-23°C, humidity, 50%, 12-hours dark/light cycle) at the Experimental Animal Center of the First Clinical College of Wuhan University. There were two mice per cage. All mice were allowed to drink and eat freely. The project was approved by our Laboratory Animal Committee, and all procedures were performed following the Guide for the Care and Use of Laboratory Animals published by the National Institutes of Health.

The I/R model was referred to our previous reports [43, 44]. Mice were completely anesthetized with sodium pentobarbital (50 mg/kg, i.p.) and placed on a thermostatic table to maintain core body temperature at 37°C. All mice were then subjected to midline dissection, followed by right nephrectomy. Next, ischemia of the left kidney was performed for 45 minutes using a noninvasive vascular clamp, followed by removal of the vascular clamp and suturing of the abdominal incision. Mice were injected intraperitoneally with 0.5 mL PBS to maintain fluid balance. After 24 hours of reperfusion, mice were executed by intraperitoneal injection of sodium pentobarbital (50 mg/kg), and renal tissues and blood were collected immediately. All mice were randomly divided into different treatment groups (*n* = 5 per group). In the Sham group, only the right kidney was removed. The left kidney in the IR group was vascularly clamped for 45 minutes; then, the clamp was released for reperfusion. The surgical operation in the IR + Fx (HPLC ≥ 95%, Sigma, F6932) group was the same as that in the IR group, but the mice were injected intraperitoneally with fucoxanthin (25 mg/kg, 50 mg/kg, and 100 mg/kg) for 24 hours before surgery. The surgical operation of the IR + Fx + EX527 (Selective Sirt1 Inhibitor, Abcam, Ab141506) group was the same as that of the IR group, except that mice were injected intraperitoneally with fucoxanthin (100 mg/kg) and EX527 (5 mg/kg) for 24 hours before surgery. The vector control group (*n* = 5) was administered equal amounts of DMSO in the vector solution.

### 2.2. Renal Function

After in vivo reperfusion, fresh blood samples were collected from mice, centrifuged at 3500 r/min for 15 minutes, and the supernatant was collected. Serum levels of urea nitrogen (BUN) and creatinine (Cr) were assessed using the Creatinine and Urea Commercial Kit (Nanjing Jiancheng Bioengineering Institute, Nanjing, China) according to the instructions provided by the manufacturer.

### 2.3. Kidney Histological Staining

For histological examination, kidneys were fixed in 4% paraformaldehyde, paraffin-embedded, sectioned at 4 *μ*m thickness, and stained with hematoxylin and eosin (H&E). The renal tubular injury was scored on a scale of 0-4 according to the percentage of tubules with necrosis, dilatation, tubular formation, and cytolysis: 0, no injury; 1, 5%-25%; 2, 25%-50%; 3, 50%-75%; and 4, >75%. The morphological assessment was performed by two experienced renal pathologists who were unaware of the experiment.

### 2.4. Immunohistochemistry

Immunohistochemical staining was performed using a commercial chemistry kit (Polink-1 one-step polymer detection system; ZSGB-BIO, Beijing, China). Sections (4 *μ*m thickness) were incubated with anti-KIM-1 antibody (Abcam, Ab78494) and anti-cleaved caspase-3 antibody (Cell Signaling, #9661) overnight at 4°C, then incubated with secondary antibody for 30 minutes at 37°C, followed by the addition of a stain. Under a microscope, five different fields of view (×400) were randomly selected to assess staining intensity. For quantification, the relative mean integrated optical density (IOD) of each group was divided by the mean IOD of the control, as determined by Image-Pro Plus 7.0 (Media Cybernetics, Rockville, MD, USA). All sections were photographed at a magnification of 400×.

### 2.5. TUNEL Staining Analysis

Apoptosis in tissues was detected using the TUNEL kit (Beyotime Biotechnology, C1089) using the TUNEL (terminal deoxynucleotidyl transferase-mediated end-of-cut labeling) staining method. Briefly, kidney tissue paraffin sections were dewaxed and rehydrated; tissues were treated with proteinase K for 20 minutes at 37°C and then washed twice with PBS. TUNEL assay solution was added dropwise and placed in a dark humidified chamber at 37°C for 1 hour. The nuclei were stained using DAPI 5 minutes before observation.

### 2.6. Western Blot Analysis

Protein levels of Bax, Bcl-2, cleaved-caspase-3, SOD1, Sirt1, Nrf2, and HO-1 were measured by protein blot analysis in HK-2 cells and kidney tissues according to standard protocols (Supplementary Figure S2-S7). HK-2 cells and kidney tissues were lysed with RIPA buffer (Beyotime Biotechnology, P0013B, China) containing protease inhibitors to obtain total protein. GAPDH was the internal loading control for the proteins. Protein content was determined by the BCA protein assay kit (Beyotime Biotechnology, P0006, China). Protein samples were separated on 10% SDS polyacrylamide gels and transferred to PVDF membranes, which were closed with 5% skim milk and immunoblotted with antibodies. The following dilutions of antibodies were used: Sirt1 (1 : 2000, Abcam, Ab12193), cleaved caspase-3 (1 : 1000, Cell Signaling, #9661), Bax (1 : 200, Cell Signaling, #2772), Bcl-2 (1 : 1000, Abcam, Ab196495), Nrf2 (1 : 1000, Abcam, Ab137550), HO-1 (1 : 2000, Abcam, Ab13243), SOD1 (1 : 5000, Abcam, Ab13498), and GAPDH (1 : 5000, PROTEINTECH NORTH AMERICA, 10494-1-AP). After incubation with appropriate secondary antibodies, protein blots were observed using chemiluminescent HRP substrates (Millipore, Billerica, MA, USA). Data analysis was performed using Image-Pro Plus 7.0 (Media Cybernetics, Rockville, MD, USA) to quantify protein levels.

### 2.7. Cell Culture and Establishment of HK-2 Cells H/R Model

The human renal proximal tubular epithelial cell line (HK-2) was provided by the Typical Culture Collection, U.S.A. HK-2 cells were incubated in Dulbecco's Modified Eagle's medium (DMEM) (Invitrogen, USA) at 37°C, including nonessential amino acids, 0.05 mg/ml bovine pituitary extract, 50 ng/ml human recombinant epidermal growth factor, 100 U/mL penicillin, 100 *μ*g/mL streptomycin, and 10% fetal bovine serum (FBS) at 5% carbon dioxide (CO_2_) and 95% air. For fucoxanthin treatment, cells were pretreated with 0.1, 1, or 5 *μ*M fucoxanthin for 24 hours. The HK-2 cell hypoxia/reoxygenation (H/R) model was referred to our previous reports [43, 44]. Briefly, HK-2 cells were exposed to hypoxia (37°C, 1% O_2_, 94% N_2_, and 5% CO_2_) in a nutrient-free (i.e., glucose-free and serum-free) medium for 12 hours to induce hypoxic injury. Subsequently, the medium was renewed, and the plates were placed in a normal cell culture incubator (5% CO_2_ and 95% air) and reoxygenated for 6 hours according to the experimental design. Controls were incubated in a conventional incubator (5% CO_2_ and 95% air).

### 2.8. Flow Cytometry

Apoptosis was assessed using Annexin V-FITC/PI apoptosis kit (Multi Sciences Biotech Co., Ltd., AP101-01, China) according to the instructions. HK-2 cells with different pretreatments were washed twice with PBS, and 1 − 10 × 10^5^ cells (including those in culture supernatant) were collected. Cells were resuspended with 500 *μ*l of 1 × Binding Buffer. Add 5 *μ*l Annexin V-FITC and 10 *μ*l PI to each tube of cells and incubate for 5 minutes at room temperature in the dark. Apoptotic cells were detected by CytoFLEX (Beckman Coulter Biotechnology (Suzhou) Co., Ltd., China).

### 2.9. Immunofluorescence

For cells, the cells of different treatments were fixed with 4% paraformaldehyde for 15 minutes according to the experimental requirements; for tissues, paraffin sections were dewaxed and hydrated. The sections were then permeabilized with 0.5% TritonX-100 for 10 minutes at room temperature. After washing with PBS, they were then closed with 10% goat serum for 1 hour at room temperature and incubated with rabbit polyclonal antibody against Sirt1/Nrf2 (1 : 200) overnight at 4°C in a refrigerator. The next day, use a secondary antibody (goat anti-rabbit) and incubate in the dark at room temperature for 1 hour. Cell nuclei were labeled by using DAPI 5 minutes before observation.

### 2.10. Small Interfering RNA (siRNA) Transfection

Sirt1-specific siRNA was used to transfect HK-2 cells for 6 hours. Meanwhile, nontargeting siRNA (Santa Cruz Biotechnology, Santa Cruz, CA, USA) was used to transfect HK-2 cells for 48 hours as a negative control. The concentration of all transfected siRNA was 100 *μ*M. After transfection, cells were cultured in DMEM/F12 containing 0.2% FBS for 48 hours. Subsequently, protein blotting was used to determine the effect of siRNA transfection.

### 2.11. Cell Viability Assay

Cell viability was determined using the Cell Counting Kit-8 (Beyotime Biotechnology, C0037, China). Briefly, HK-2 cells were inoculated into 96-well plates. After 24 hours incubation, cells were incubated with different concentrations of fucoxanthin (0, 0.1, 1, 5, 10, 20, and 50 *μ*M) for 24 hours. Cells were then incubated with CCK-8 reagent for 60 minutes at 37°C. The optical density values were detected at 450 nm (Molecular Devices, USA).

### 2.12. Measurement of ROS Level

Intracellular ROS levels were measured according to the instructions of the Reactive Oxygen Species Detection Kit (Beyotime Biotechnology, S0033S, China). Briefly, HK-2 cells pretreated with different reagents were incubated with 10 *μ*M of dichlorodihydrofluorescein diacetate (DCFH-DA) for 20 minutes at 37°C in the dark. For tissues, fresh tissues were frozen sectioned, and then intratissue ROS levels were measured using the Dihydroethidium kit (Beyotime Biotechnology, S0063, China). Frozen sections of fresh kidneys from differently treated mice were incubated with 10 *μ*M of Dihydroethidium at 37°C for 30 minutes in the dark. Cell nuclei were labeled by using DAPI 5 minutes before observation.

### 2.13. Statistical Analysis

All data were expressed as mean ± standard error of the mean (SEM). Statistical analysis involved one-way analysis of variance (ANOVA) and the Student-Newman-Keuls test. Differences were considered statistically significant when *p* < 0.05.

## 3. Results

### 3.1. Fucoxanthin Ameliorated Renal Dysfunction and Tissue Structural Damage in Mice after Renal I/R

**Figure 1:**
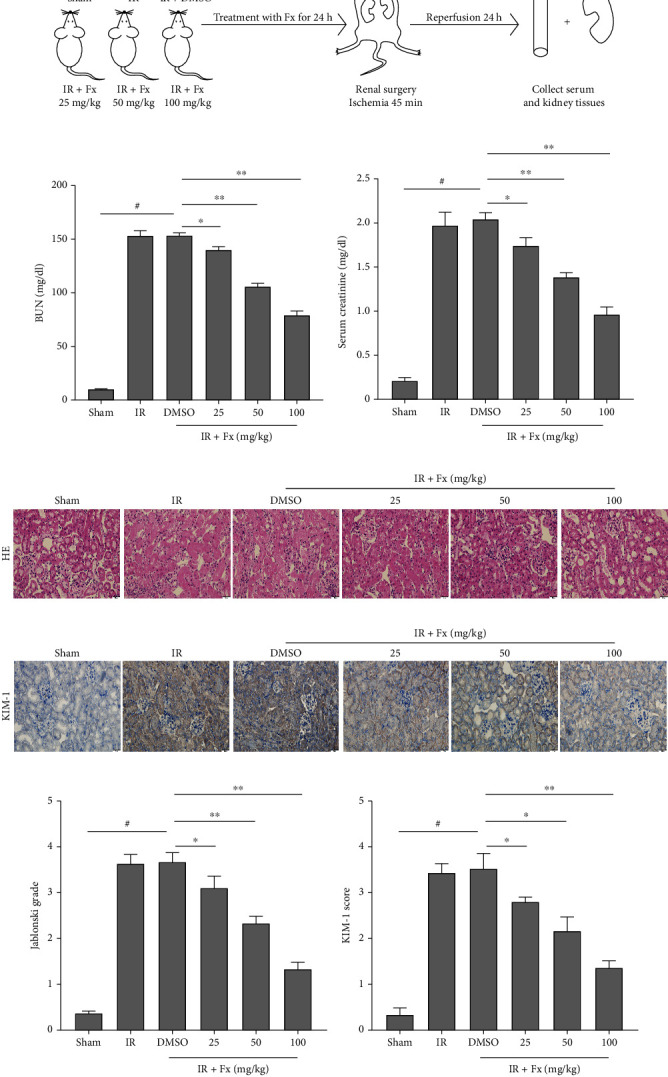
Pretreatment fucoxanthin decreased BUN and Cr levels, attenuated pathological injury, and reduced KIM-1 expression in mice after renal I/R. The I/R model was established with 45 minutes of ischemia and 24 hours of reperfusion. The doses administered in the fucoxanthin treatment groups were 25 mg/kg, 50 mg/kg, and 100 mg/kg. (a) Schematic diagram of the experiment process. (b) Serum BUN levels. (c) Serum Cr levels. (d) and (f) Representative images of kidney HE staining (magnification ×400; scale bars = 20 *μ*m) and its quantitative analysis. (e) and (g) Representative images of KIM-1 immunohistochemical staining (magnification ×400; scale bars = 20 *μ*m) and its quantitative analysis. Values are expressed as mean ± SEM, *n* = 5. ^#^*p* < 0.05 compared with Sham group, ^∗^*p* < 0.05 and ^∗∗^*p* < 0.01 compared with IR + DMSO group.

The experimental flow diagram was shown in Figure 1(a). In the present experiment, we established an I/R model and confirmed the protective effect of fucoxanthin on renal I/R in mice. Compared with the sham group, I/R resulted in obvious renal dysfunction, as evidenced by a significant increase in serum BUN and serum Cr levels. In the treatment group, pretreatment with fucoxanthin remarkably restored some of the renal impairment and showed a dose-dependence (Figures 1(b) and 1(c)). In addition, H&E staining showed that I/R induced significant renal parenchymal injury, mainly manifested by massive tubular epithelial cell necrosis, tubular dilatation, and tubular pattern formation. 100 mg/kg fucoxanthin pretreatment dramatically reduced tubular injury and mainly preserved normal renal tissue structure (Figures 1(d) and 1(f)). Immunohistochemistry showed high expression of KIM-1 (Kidney Injury Molecule-1) in the kidney tissue of I/R mice compared to the sham group, and fucoxanthin eliminated the elevated KIM-1 expression (Figures 1(e) and 1(g)).

### 3.2. Fucoxanthin Dramatically Attenuated Oxidative Stress-Induced Apoptosis in Mice after Renal I/R

**Figure 2:**
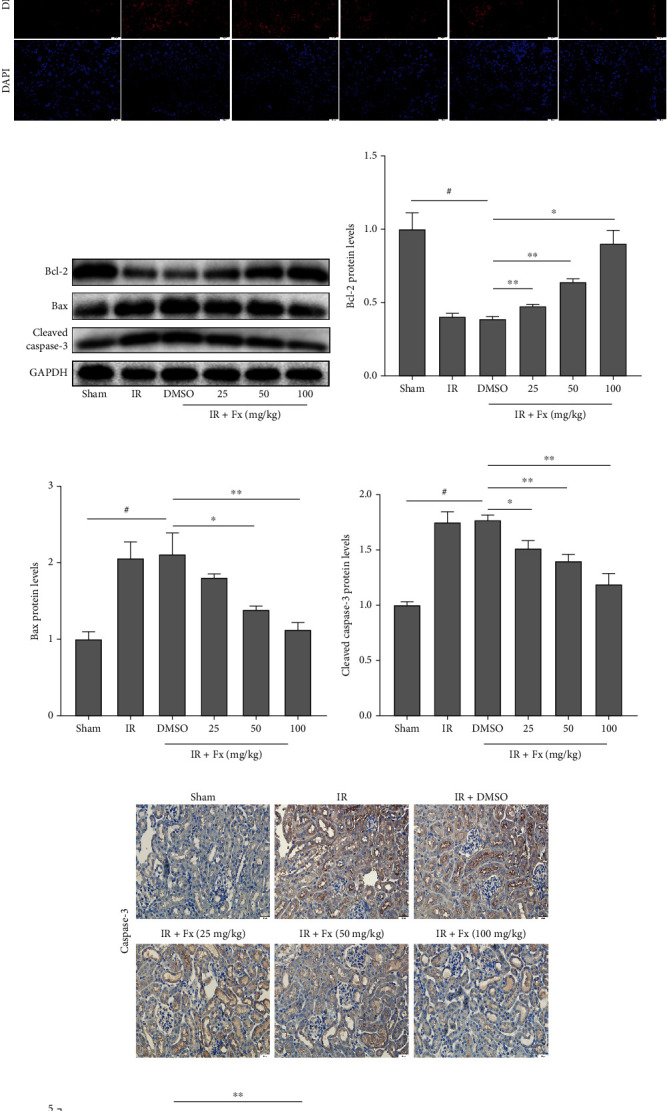
Fucoxanthin inhibited ROS levels and attenuated apoptosis in mice after renal ischemia-reperfusion. The I/R model was established with 45 minutes of ischemia and 24 hours of reperfusion. The doses administered in the fucoxanthin treatment groups were 25 mg/kg, 50 mg/kg, and 100 mg/kg. (a) and (b) SOD1 protein expression. (c) and (d) Representative images of DHE staining (magnification × 400; scale bars = 20 *μ*m) and its quantitative analysis. (e)–(h) Expression of apoptosis-related proteins Bcl-2, Bax, cleaved caspase-3, and their quantitative analysis. (i)–(j) Representative images of caspase-3 immunohistochemistry (magnification ×400; scale bars = 20 *μ*m) and their quantitative analysis. (k)–(l) Representative images of TUNEL staining (magnification ×400; scale bars = 20 *μ*m) and their quantitative analysis. Apoptotic cells were detected by TUNEL (red). Values are expressed as mean ± SEM, *n* = 3. ^#^*p* < 0.05 compared with Sham group, ^∗^*p* < 0.05 and ^∗∗^*p* < 0.01 compared with IR + DMSO group.

Oxidative stress is an important pathway of renal injury caused by I/R and is known to induce apoptosis in kidney cells. Compared with the sham group, I/R injury significantly decreased renal SOD1 protein expression, while fucoxanthin pretreatment markedly upregulated renal SOD1 protein expression levels after I/R (Figures 2(a) and 2(b)). In addition, we reflected the ROS level in the kidney tissue by detecting DHE. As shown, renal I/R could significantly increase DHE fluorescence intensity compared to the sham group, while fucoxanthin pretreatment dramatically decreased DHE fluorescence intensity in a dose-dependent manner (Figures 2(c) and 2(d)).

Our previous study demonstrated that oxidative stress in I/R injury leads to severe apoptosis, whereas the oxidative stress inhibitor NAC inhibits oxidative stress and reduces apoptosis [43]. Therefore, to further evaluate the effect of fucoxanthin on apoptosis after renal I/R, we examined the expression of a series of apoptosis-related proteins. As expected, renal I/R injury significantly decreased the levels of the apoptosis-protecting protein Bcl-2 and significantly upregulated the levels of the apoptosis-promoting proteins Bax and cleaved caspase-3 compared to the sham group. However, pretreatment of mice with fucoxanthin reversed the changes in the levels of apoptosis-related proteins (Figures 2(e)–2(h)). In addition, the results of immunohistochemical detection of caspase-3 were consistent with the results of western blotting (Figures 2(i) and 2(j)). In the TUNEL staining results, the number of apoptosis-positive cells in the kidney was significantly increased after I/R compared with the sham group, and pretreatment of mice with fucoxanthin significantly reduced the number of TUNEL-positive cells (Figures 2(k) and 2(l)). These results suggested that fucoxanthin could effectively attenuate apoptosis caused by oxidative stress in the mouse kidney after I/R.

### 3.3. In Vitro, Fucoxanthin Suppressed Apoptosis Caused by Oxidative Stress after H/R

**Figure 3:**
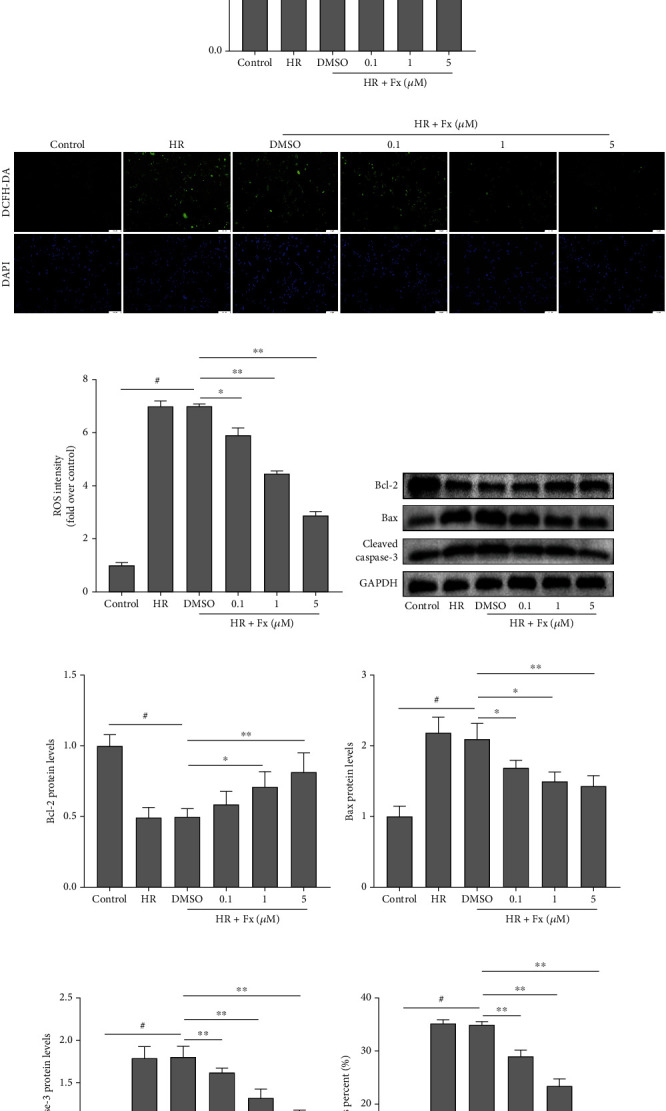
Fucoxanthin attenuated ROS levels and apoptosis in HK-2 cells after H/R injury. The H/R model was established by hypoxia for 12 hours and reoxygenation for 6 hours. The HK-2 cells were treated with fucoxanthin (0.1 *μ*M, 1 *μ*M, and 5 *μ*M) for 24 hours and then experienced H/R. (a) Cytotoxicity of fucoxanthin on HK-2 cells. (b) and (c) SOD1 protein expression. (d)–(e) Representative images of DCFH-DA staining (magnification ×100; scale bars = 100 *μ*m) and their quantitative analysis. (f)–(i) Expression of apoptosis-related proteins Bcl-2, Bax, cleaved caspase-3, and their quantitative analysis. (j) and (k) Percentage of apoptotic cells detected by flow cytometry. Values are expressed as mean ± SEM, *n* = 3. ^#^*p* < 0.05 compared with control group, ^∗^*p* < 0.05 and ^∗∗^*p* < 0.01 compared with HR + DMSO group.

To investigate the effect of fucoxanthin on HK-2 cells exposed to H/R conditions, we first performed CCK-8 to determine the optimal concentration of fucoxanthin. After pretreatment of HK-2 cells with fucoxanthin for 24 h under normoxic conditions, the viability of HK-2 cells decreased to 85% when the concentration reached 10 *μ*M, and fucoxanthin was significantly toxic to cells. Therefore, we determined fucoxanthin concentrations of 0.1, 1, and 5 *μ*M (Figure 3(a)). Subsequently, we further examined the effect of fucoxanthin on the oxidative stress of HK-2 cells after undergoing H/R. As shown, the expression level of SOD1 protein was decreased in HK-2 cells after exposure to H/R compared to the control group, while fucoxanthin pretreatment significantly upregulated the expression level of SOD1 protein (Figures 3(b) and 3(c)). Similarly, H/R markedly upregulated ROS levels in HK-2 cells, while fucoxanthin effectively reversed the changes in ROS (Figures 3(d) and 3(e)).

In vitro, we further verified the effect of fucoxanthin on oxidative stress-induced apoptosis. Compared with the control group, Bcl-2 protein expression was markedly decreased in HK-2 cells of the H/R group, but Bax and cleaved caspase-3 protein expression was significantly increased. And the opposite protein expression change was observed by pretreatment with fucoxanthin compared with the HR + DMSO group (Figures 3(f)–3(i)). In addition, the results of flow cytometry to detect apoptosis rate were also consistent with the results of western blotting. And 5 *μ*M of fucoxanthin had the strongest antiapoptotic effect (Figures 3(j) and 3(k)). Therefore, the fucoxanthin concentration was 5 *μ*M in all subsequent in vitro experiments.

### 3.4. Sirt1/Nrf2/HO-1 Signaling Pathway in HK-2 Cells Was Activated by Fucoxanthin after H/R

**Figure 4:**
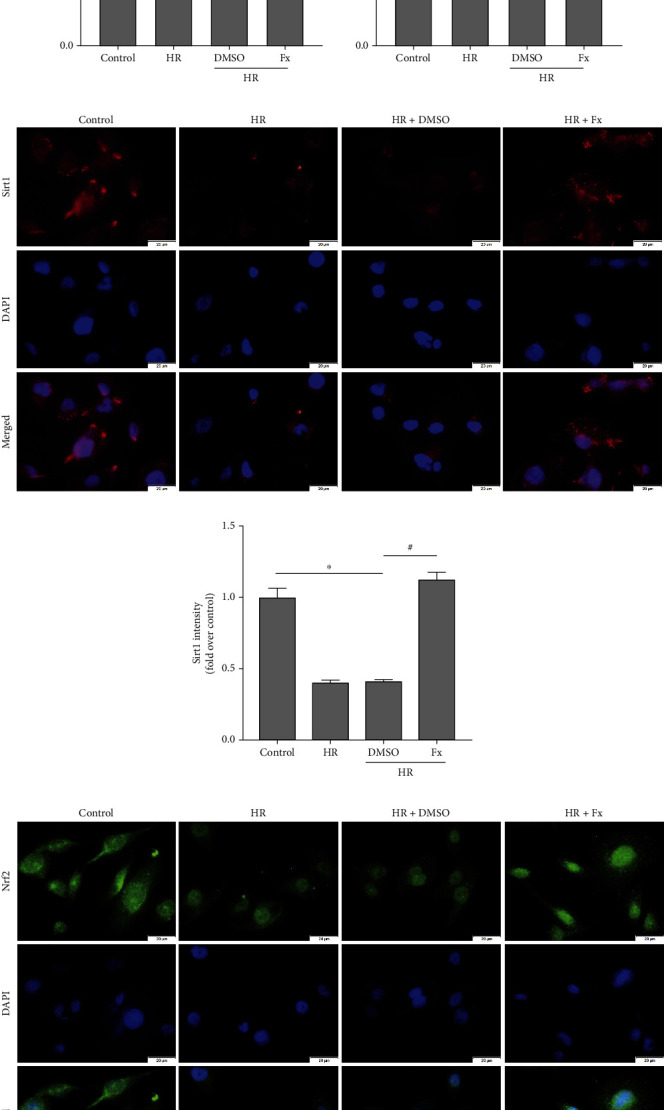
Sirt1, Nrf2, and HO-1 protein expression levels were upregulated by fucoxanthin. The H/R model was established by hypoxia for 12 hours and reoxygenation for 6 hours. The HK-2 cells were treated with 5 *μ*M fucoxanthin for 24 hours and then experienced H/R. (a)–(d) Sirt1, Nrf2, and HO-1 protein expression and their quantitative analysis. (e) and (f) Representative images of Sirt1 immunofluorescence staining (magnification ×400; scale bars = 20 *μ*m) and their quantitative analysis. (g) and (h) Representative images of Nrf2 immunofluorescence staining (magnification ×400; scale bars = 20 *μ*m) and its quantitative analysis of cytoplasmic and nuclear expression. Values are expressed as mean ± SEM, *n* = 3. ^∗^*p* < 0.05 compared with control group, ^#^*p* < 0.05 compared with HR + DMSO group.

To further explore the potential mechanisms that may constitute fucoxanthin's resistance to oxidative stress and attenuation of apoptosis in HK-2 cells, we examined a series of related proteins. We found that the expression level of Sirt1 protein was significantly decreased when HK-2 cells were exposed to H/R compared to the control group, and the expression levels of Nrf2 and its downstream HO-1 were also significantly decreased. In contrast, fucoxanthin pretreatment markedly upregulated the expression of Sirt1 in HK-2 cells after H/R, and the protein levels of Nrf2 and HO-1 were also upregulated by fucoxanthin (Figures 4(a)–4(d)). In addition, immunofluorescence of HK-2 cells showed that fucoxanthin pretreatment significantly enhanced the red fluorescence intensity of Sirt1 protein in HK-2 cells after H/R, which was more visually demonstrated by semiquantitative analysis (Figures 4(e) and 4(f)). In addition, interestingly, we found that nuclear translocation of Nrf2 may be crucial for fucoxanthin to inhibit oxidative stress. As shown in the figure, in the control group, the green fluorescence intensity of Nrf2 was higher, and it was expressed in both cytoplasm and nucleus; while in the HR and HR + DMSO groups, the green fluorescence intensity of Nrf2 was significantly lower, and the expression of Nrf2 was confined to the nucleus; however, fucoxanthin pretreatment dramatically upregulated the green fluorescence intensity of Nrf2 and further promoted nuclear translocation of Nrf2 (Figures 4(g) and 4(h)). These data suggested that fucoxanthin may promote Nrf2 nuclear translocation and activate its downstream HO-1 to resist H/R-induced oxidative stress and apoptosis by activating Sirt1.

### 3.5. Inhibition of Sirt1 Reversed the Protective Effect of Fucoxanthin on HK-2 Cells In Vitro

**Figure 5:**
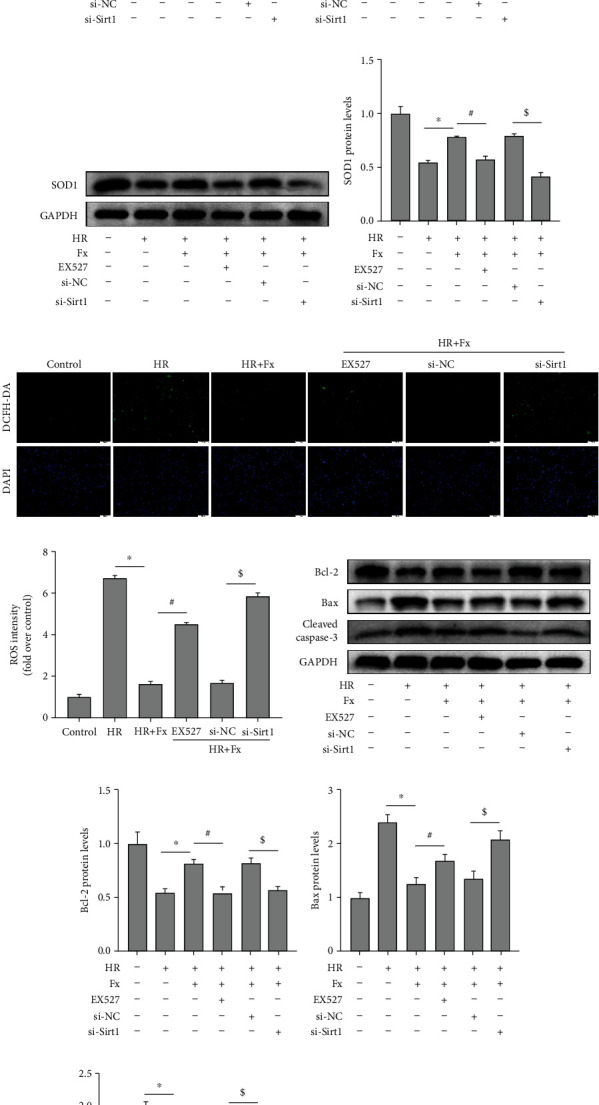
Inhibition of Sirt1 reversed the role of fucoxanthin in protecting HK-2 cells from H/R injury. The H/R model was established by hypoxia for 12 hours and reoxygenation for 6 hours. The HK-2 cells were treated with 5 *μ*M fucoxanthin, 20 *μ*M EX527, si-NC, or si-Sirt1 for 24 hours and then experienced H/R. (a)–(d) Sirt1, Nrf2, and HO-1 protein expression and their quantitative analysis. (e) and (f) SOD1 protein expression and its quantitative analysis. (g) and (h) Representative images of DCFH-DA staining (magnification ×100; scale bars = 100 *μ*m) and their quantitative analysis. (i)–(l) Expression of apoptosis-related proteins Bcl-2, Bax, cleaved caspase-3, and their quantitative analysis. (m) and (n) Percentage of apoptotic cells detected by flow cytometry. Values are expressed as mean ± SEM, *n* = 3.^∗^*p* < 0.05 compared with HR group, ^#^*p* < 0.05 compared with HR + Fx group, ^$^*p* < 0.05 compared with HR + Fx + si − NC group.

To further elucidate the significance of Sirt1 in the antiapoptotic effect of fucoxanthin, we used a selective inhibitor of Sirt1, EX527, and siRNA to suppress Sirt1 expression. As expected, EX527 could abrogate the upregulation of Sirt1 by fucoxanthin to some extent, and the effect of Sirt1 knockdown by si-Sirt1 was more pronounced. In addition, protein expression of Nrf2 and HO-1 in fucoxanthin-pretreated HK-2 cells was also significantly downregulated by EX527 and si-Sirt1 (Figures 5(a)–5(d)).

Subsequently, we further explored the effect of Sirt1 inhibition on oxidative stress and apoptosis. Protein expression of SOD1 was markedly increased by fucoxanthin compared to the H/R group, and then this increase was significantly suppressed by EX527 and si-Sirt1 (Figures 5(e) and 5(f)), and the inhibition of ROS by fucoxanthin was greatly inhibited by EX527 and si-Sirt1 (Figures 5(g) and 5(h)). In addition, nhibition of Sirt1 significantly inhibited the antiapoptotic ability of fucoxanthin, as shown in the figure, inhibition of Sirt1 significantly reduced the expression of apoptosis-protective protein Bcl-2 and increased the expression of proapoptotic proteins Bax and cleaved caspase-3 (Figures 5(i)–5(l)). Meanwhile, the HK-2 cell apoptosis rate was also significantly upregulated by EX527 and si-Sirt1 (Figures 5(m) and 5(n)).

### 3.6. In Vivo, Fucoxanthin Upregulated Sirt1/Nrf2/HO-1 Protein Expression in Mice after Renal I/R

**Figure 6:**
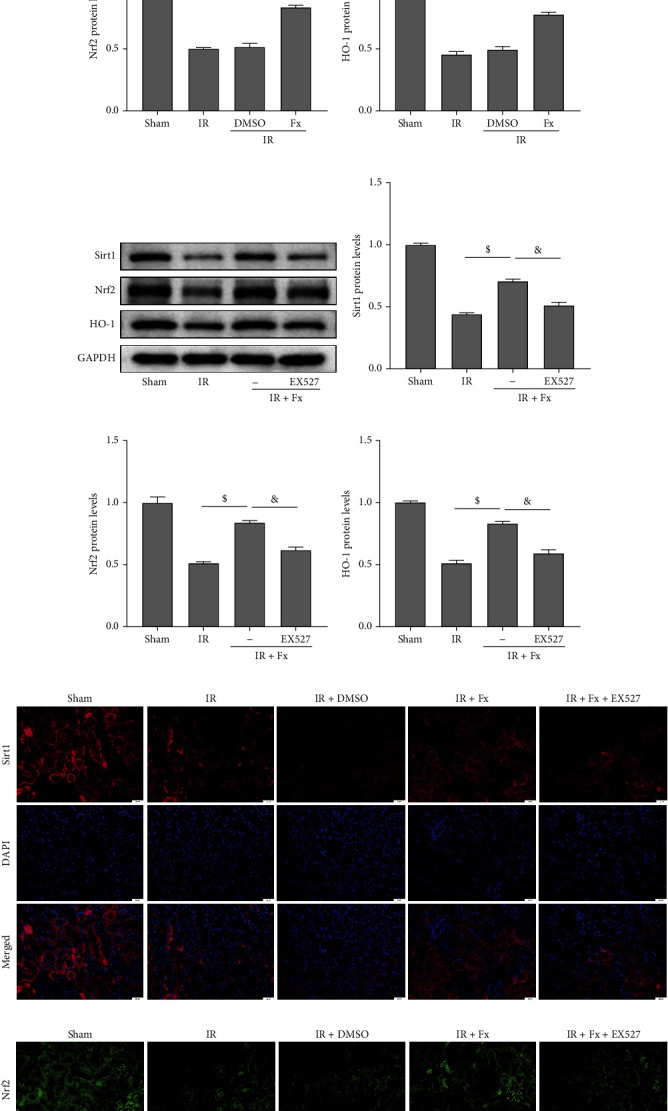
Fucoxanthin upregulated Sirt1, Nrf2, and HO-1 protein expression after renal I/R injury in mice, and EX527 suppressed the effect of fucoxanthin. The I/R model was established with 45 minutes of ischemia and 24 hours of reperfusion. Mice were treated with 100 mg/kg of fucoxanthin or 5 mg/kg of EX527. (a)–(d) Effect of fucoxanthin on Sirt1, Nrf2, and HO-1 protein expression and their quantitative analysis. (e)–(h) Effect of coadministration of fucoxanthin and EX527 on Sirt1, Nrf2, HO-1 protein expression, and their quantitative analysis. (i) Representative images of immunofluorescence staining of Sirt1 in kidney tissues (magnification × 400; scale bars = 20 *μ*m). (j) Representative images of immunofluorescence staining of Nrf2 in kidney tissues (magnification ×400; scale bars = 20 *μ*m). Values are expressed as mean ± SEM, *n* = 3. ^∗^p < 0.05 compared with Sham group, ^#^*p* < 0.05 compared with IR + DMSO group, ^$^*p* < 0.05 compared with IR group, ^&^*p* < 0.05 compared with IR + Fx group.

Compared with the sham group, renal Sirt1 protein expression levels were significantly decreased by I/R, and there was no significant difference between the IR and IR + DMSO groups. In contrast, pretreatment with fucoxanthin dramatically upregulated Sirt1 protein expression in the kidneys of mice. In ddition, when mice underwent renal I/R, the expression levels of Nrf2 and HO-1 proteins in the kidney were significantly decreased, and fucoxanthin substantially reversed this change (Figures 6(a)–6(d)). The results of immunofluorescence detection of Sirt1 and Nrf2 in kidney tissues were also consistent with the results of western blotting (Figures 6(i) and 6(j)).

### 3.7. Coadministration of EX527 and Fucoxanthin Promoted Oxidative Damage Caused by Renal I/R in Mice

We then proceeded to suppress Sirt1 in vivo to explore the mechanism by which fucoxanthin attenuates renal I/R injury. Combined pretreatment of mice with fucoxanthin and EX527 markedly downregulated Sirt1 protein expression and significantly suppressed Nrf2 and HO-1 protein expression levels compared to the fucoxanthin-treated group (Figures 6(e)–6(h)). Immunofluorescence of kidney tissues also confirmed that the ability of fucoxanthin to upregulate Sirt1 and Nrf2 protein expression levels was dramatically eliminated by EX527 (Figures 6(i) and 6(j)).

**Figure 7:**
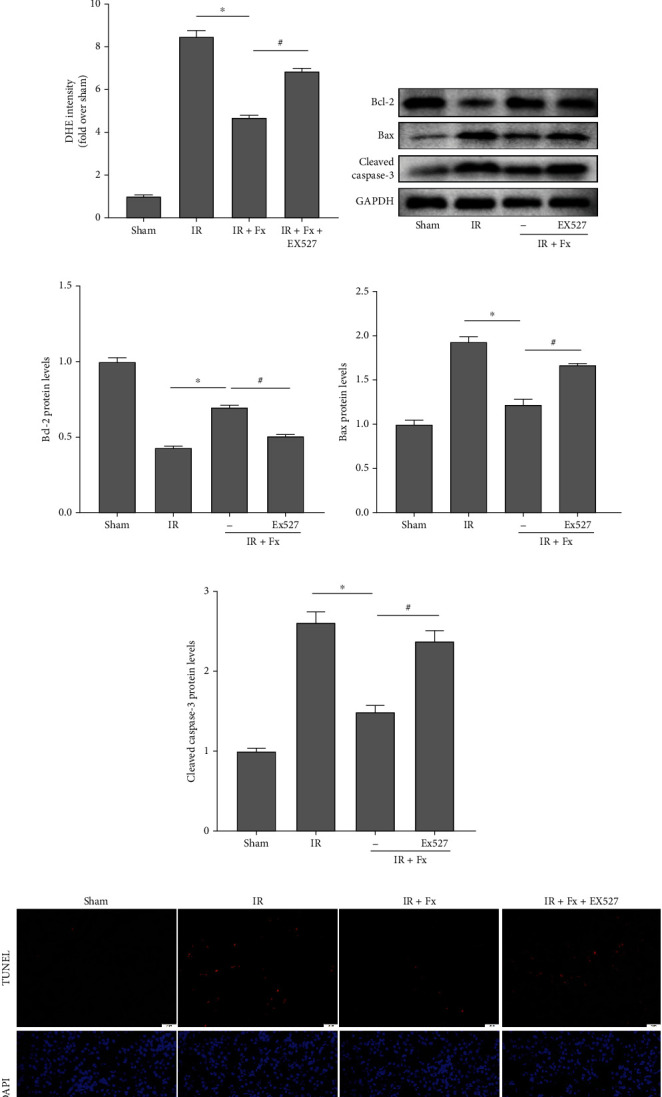
EX527 eliminated the protective effect of fucoxanthin on renal I/R injury in mice. The I/R model was established with 45 minutes of ischemia and 24 hours of reperfusion. Mice were treated with 100 mg/kg of fucoxanthin or 5 mg/kg of EX527. (a) and (b) SOD1 protein expression. (c) and (d) Representative images of DHE staining (magnification × 400; scale bars = 20 *μ*m) and their quantitative analysis. (e)–(h) Expression of apoptosis-related proteins Bcl-2, Bax, cleaved caspase-3, and their quantitative analysis. (i) and (j) Representative images of TUNEL staining (magnification × 400; scale bars = 20 *μ*m) and their quantitative analysis. Apoptotic cells were detected by TUNEL (red). Values are expressed as mean ± SEM, *n* = 3. ^∗^*p* < 0.05 compared with IR group, ^#^*p* < 0.05 compared with IR + Fx group.

Then, we examined the effect of EX527 on oxidative stress-induced apoptosis in mice kidneys after I/R. As expected, the combined use of fucoxanthin and EX527 markedly suppressed the expression of SOD1 protein (Figures 7(a) and 7(b)), while significantly upregulating the red fluorescence intensity of DHE in kidney tissues (Figures 7(c) and 7(d)). In the results of apoptosis-related proteins, EX527 dramatically decreased the expression of Bcl-2 protein and significantly upregulated the expression of Bax and cleaved caspase-3 protein compared with the fucoxanthin group (Figures 7(e)–7(h)). The ability of fucoxanthin to reduce TUNEL-positive cells was also inhibited by EX527 in the TUNEL staining results (Figures 7(i) and 7(j)). These results suggested that inhibition of Sirt1 significantly inhibited the effect of fucoxanthin in attenuating renal I/R injury in mice.

## 4. Discussion

In the present experiments, we worked to investigate the role of fucoxanthin in renal I/R injury in mice and to explore its potential mechanisms. First, we determined the role of fucoxanthin in a classical animal model of renal I/R injury. Our results showed that pretreatment with fucoxanthin attenuated renal dysfunction, reduced tissue structural damage, and attenuated oxidative stress-induced apoptosis in I/R injury in mice. Subsequently, we found that fucoxanthin pretreatment in HK-2 cells exposed to H/R similarly attenuated oxidative stress-induced apoptosis. Furthermore, we found that fucoxanthin could activate the Sirt1/Nrf2/HO-1 signaling pathway after I/R injury or H/R injury and that the protective effect of fucoxanthin could be reversed using pharmacological inhibitors of Sirt1 or si-RNA. Thus, our results suggest that fucoxanthin attenuates renal ischemia-reperfusion-induced renal injury by activating the Sirt1/Nrf2/HO-1 signaling pathway, and fucoxanthin may be a novel therapeutic agent in renal ischemic injury.

Acute kidney injury is a clinical syndrome characterized by a sudden decrease in the glomerular filtration rate with high morbidity and mortality [45]. Approximately 7% of hospitalized patients and nearly 35% of patients in intensive care suffer from AKI. In critically ill patients, AKI mortality may exceed 50% [46]. The poor prognosis of AKI is associated with chronic kidney disease and end-stage renal disease [47, 48]. Renal ischemia-reperfusion injury is widely recognized as a common cause of AKI, which may be caused by various clinical factors [6]. Multiple pathological processes are leading to AKI due to renal I/R injury, including oxidative stress, inflammatory response, vascular injury, cell necrosis, and apoptosis [49]. Numerous studies have reported that oxidative stress is associated with I/R-induced AKI. The kidney produces excess ROS during blood flow reperfusion, which leads to oxidative damage to biomolecules and exacerbates ischemic tissue damage [50, 51]. Therefore, suppressing oxidative stress is a potentially effective approach to treat renal I/R injury. Fucoxanthin is a marine carotenoid with a unique chemical structure. Previous studies have shown that fucoxanthin has a strong antioxidant stress capacity to scavenge excess ROS in a variety of diseases [38, 39, 52]. In the present study, we found that pretreatment with fucoxanthin attenuated renal dysfunction and reduced tissue structural damage due to I/R injury in mice. Fucoxanthin increased SOD1 protein expression in tissues and scavenged I/R injury-induced ROS in a dose-dependent manner. Our previous studies demonstrated that oxidative stress in renal I/R injury leads to severe apoptosis [43]. We further examined the expression levels of apoptosis-related proteins in the kidneys of fucoxanthin-pretreated mice. After the occurrence of renal I/R injury, the expression of apoptosis-protective protein Bcl-2 was significantly suppressed, and the expression of apoptosis-promoting proteins Bax and cleaved caspase-3 was markedly increased in mice kidneys, while fucoxanthin pretreatment reversed the changes of several proteins. In addition, TUNEL staining results showed that fucoxanthin significantly reduced the number of apoptosis-positive cells. Similarly, we found consistent results in in vitro cell experiments. Fucoxanthin significantly attenuated oxidative stress-induced apoptosis in HK-2 cells after H/R.

Sirt1 is an NAD^+^-dependent deacetylase that plays a prominent role in many cellular activities through its deacetylation function, including gene transcription, cellular senescence, apoptosis, and energy stress [12, 13, 53]. A multitude of studies has demonstrated that in renal ischemic diseases, activation of Sirt1 significantly attenuates renal injury [23, 24]. For example, in a diabetic renal ischemia-reperfusion injury study, researchers used resveratrol to activate Sirt1 to significantly attenuate endoplasmic reticulum stress and cellular scorching induced by renal I/R injury in diabetic rats [54]. In addition, specific overexpression of Sirt1 in the kidney in AKI induced by the chemotherapeutic drug cisplatin preserved peroxisome function to prevent acute kidney injury [55]. Nrf2 is known to be a transcription factor expressed in various cell types, acting upstream of the antioxidant defense system and playing a major role in the regulation of redox homeostasis [56]. Under normal conditions, Nrf2 is mainly localized in the cytoplasm and forms a complex with Keap1, which is anchored to the actin cytoskeleton. When inducers, such as oxidizing molecules, disrupt the Keap1-Nrf2 complex, Nrf2 dissociates from Keap1 and migrates into the nucleus. In the nucleus, Nrf2 binds to the 5′-upstream regulatory antioxidant response element (ARE) regions of downstream genes (HO-1 and NQO1) and promotes their transcriptional activity [57, 58]. Recent studies suggest that Sirt1 may be an upstream target for regulating Nrf2-ARE and activation of Sirt1 could act on Nrf2 through its deacetylation [59]. In addition, Sirt1 has been reported to activate the Nrf2-ARE pathway in glomerular thylakoid cells [29]. The current study suggests that fucoxanthin is a potent Sirt1 activator. For example, fucoxanthin mitigates high glucose-induced oxidative stress and inhibits renal fibrosis via the Akt/Sirt1/FoxO3*α* signaling pathway [60]. In one research on subarachnoid hemorrhage, fucoxanthin reduced oxidative damage through a Sirt1-dependent pathway [39]. In addition, fucoxanthin activates the Sirt1/AMPK signaling pathway to inhibit lipid accumulation in hepatocytes [61]. In our research, we found that I/R or H/R injury resulted in a remarkable decrease in Sirt1 expression, while fucoxanthin significantly increased Sirt1 expression. In addition to this, we examined the protein expression levels of transcription factor Nrf2 and its downstream HO-1. We found that the expression of Nrf2 and HO-1 was significantly downregulated after the occurrence of I/R or H/R injury, while fucoxanthin significantly upregulated the protein expression levels of Nrf2 and HO-1. In addition, immunofluorescence of HK-2 cells revealed that fucoxanthin promoted Nrf2 nuclear translocation. Subsequently, we inhibited Sirt1 in vivo and in vitro using EX527, a selective inhibitor of Sirt1 or si-Sirt1 and found that the expression of Nrf2 and HO-1 was also significantly suppressed, and the inhibitory effect of fucoxanthin on oxidative stress and reduction of apoptosis was dramatically reversed by Sirt1 suppression.

Oxidative stress is one of the major factors in the pathogenesis of ischemic diseases including renal I/R injury, where excessive oxidative stress leads to mitochondrial dysfunction, abnormal energy metabolism, and ultimately apoptosis [62]. Carotenoids are natural antioxidants that are widely available in nature and have great advantages in the treatment of oxidative stress-related diseases [32]. The studies showed that fucoxanthin can effectively inhibit oxidative stress and exert biological activity as an agonist of Sirt1 or Nrf2 in diseases such as ischemic stroke, subarachnoid hemorrhage, alcoholic liver injury, and renal fibrosis [38, 39, 58, 63]. In addition, another marine carotenoid: astaxanthin, which is found mainly in salmon, crab, shrimp, and seaweed, is known for its powerful antioxidant and anti-inflammatory properties [64]. Unlike plants, animals and humans cannot synthesize carotenoids and must obtain them through diet [32]. Therefore, edible carotenoids have the potential advantage to become a new antioxidant therapy. In conclusion, our study suggests that fucoxanthin attenuates oxidative damage by scavenging overproduced ROS in renal ischemia-reperfusion injury and that the mechanism may be related to the activation of the Sirt1/Nrf2/HO-1 signaling pathway.

## 5. Conclusions

In summary, our results indicated that we initially revealed the protective role of the marine carotenoid fucoxanthin in renal I/R injury. Furthermore, we found that fucoxanthin inhibited oxidative stress-induced apoptosis in I/R injury through activation of the Sirt1/Nrf2/HO-1 signaling pathway. However, whether fucoxanthin acts on renal I/R injury through other mechanisms still needs to be further explored, and the specific mechanism of interaction between Sirt1 and Nrf2 also needs to be investigated in depth. In conclusion, our study suggests that fucoxanthin may become a novel drug for the treatment of renal ischemic diseases and that the Sirt1/Nrf2/HO-1 signaling pathway may become a new therapeutic target.

## Data Availability

The datasets in this study are available from the corresponding author.

## Abbreviations

AKI: Acute kidney injury
Bcl-2: B-cell lymphoma 2
BUN: Blood urea nitrogen
Cr: Creatinine
DCFH-DA: Dichlorodihydrofluorescein diacetate
DHE: Dihydroethidium
Fx: Fucoxanthin
HO-1: Heme oxygenase-1
H/R: Hypoxia/reoxygenation
HK-2: Human renal proximal tubular epithelial cell line
I/R: Ischemia/reperfusion
NAC: N-acetyl-cysteine
NQO1: NAD(P)H quinone oxidoreductase 1
Nrf2: Nuclear factor erythroid 2-related factor 2
ROS: Reactive oxygen species
siRNA: Small interfering RNA
Sirt1: Silent information regulator 1
SOD1: Superoxide dismutase 1
TUNEL: Terminal deoxynucleotidyl transferase-mediated dUTP nick end-labeling.

## Supplementary Materials

Supplementary Materials Figure S1: the chemical structure diagram of fucoxanthin. Figure S2: Western blot data of Figure 2. Figure S3: Western blot data of Figure 3. Figure S4: Western blot data of Figure 4. Figure S5: Western blot data of Figure 5. Figure S6: Western blot data of Figure 6. Figure S7: Western blot data of Figure 7.
