# Supplementary material for: Fucoxanthin Attenuates Oxidative Damage by Activating the Sirt1/Nrf2/HO-1 Signaling Pathway to Protect the Kidney from Ischemia-Reperfusion Injury
Source: Oxid Med Cell Longev. 2022 Jan 28;2022:7444430. doi: 10.1155/2022/7444430 (PMC8816562; doi:10.1155/2022/7444430)

**Supplementary materials**

**Figure S1 The chemical structure diagram of fucoxanthin.**


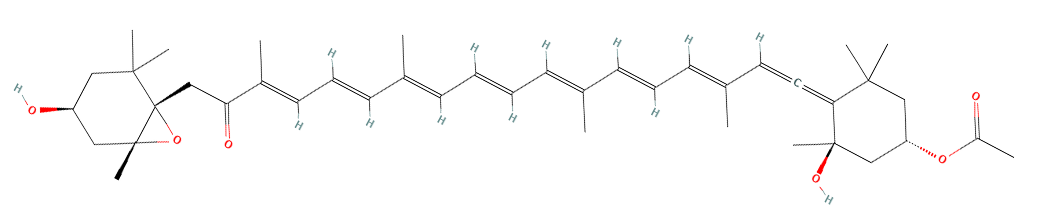


**Figure S2 Western blot data of Figure 2**
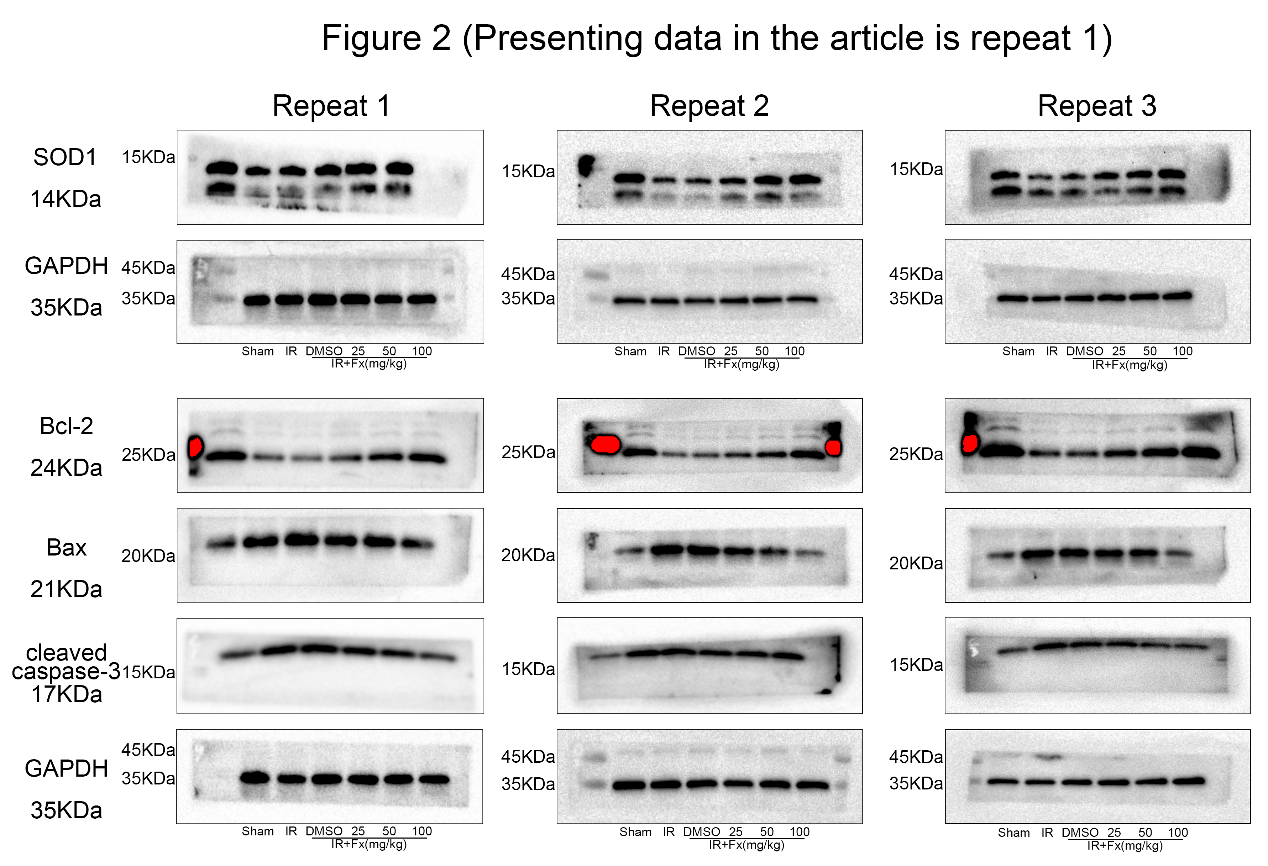


**Figure S3 Western blot data of Figure 3**
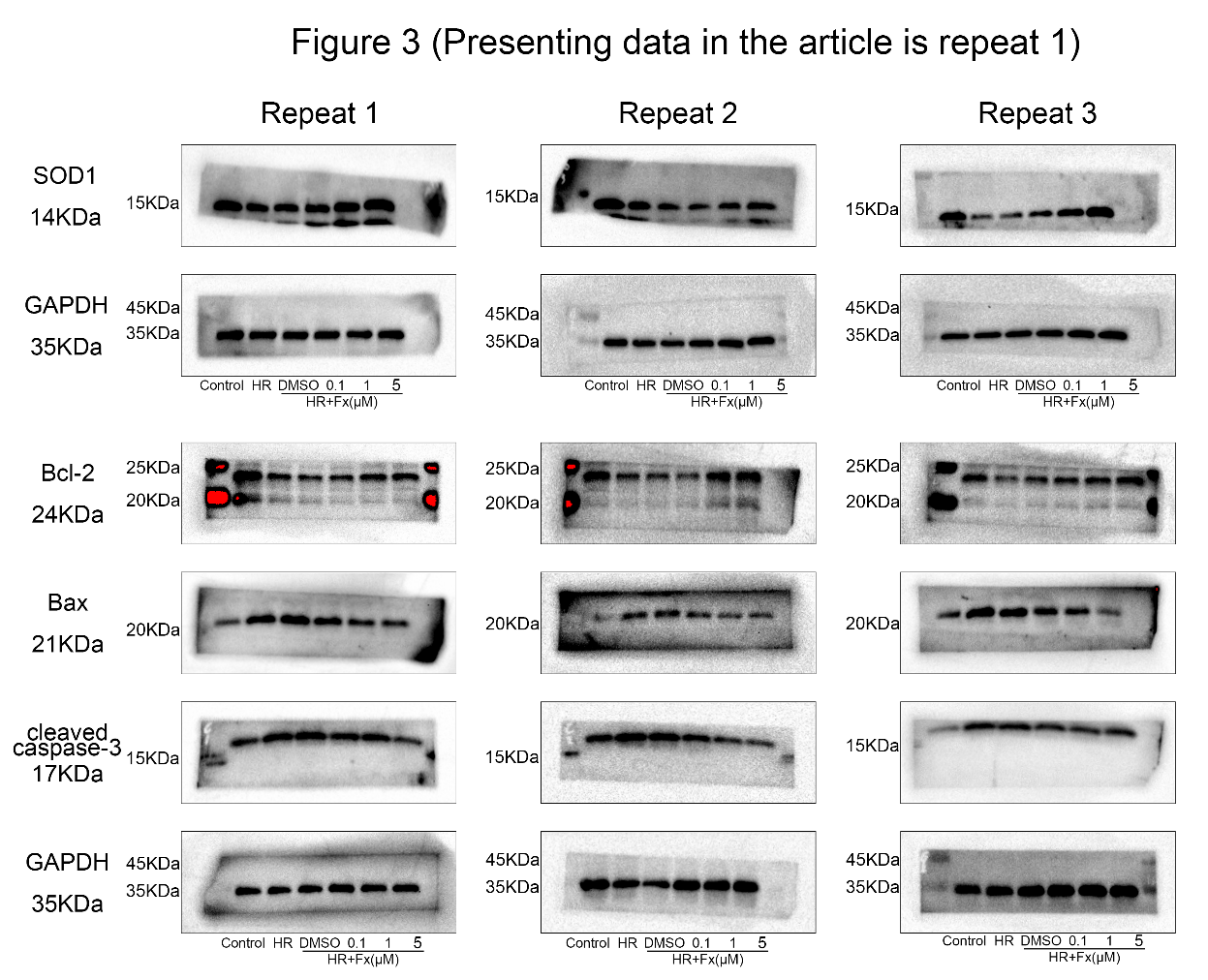


**Figure S4 Western blot data of Figure 4**
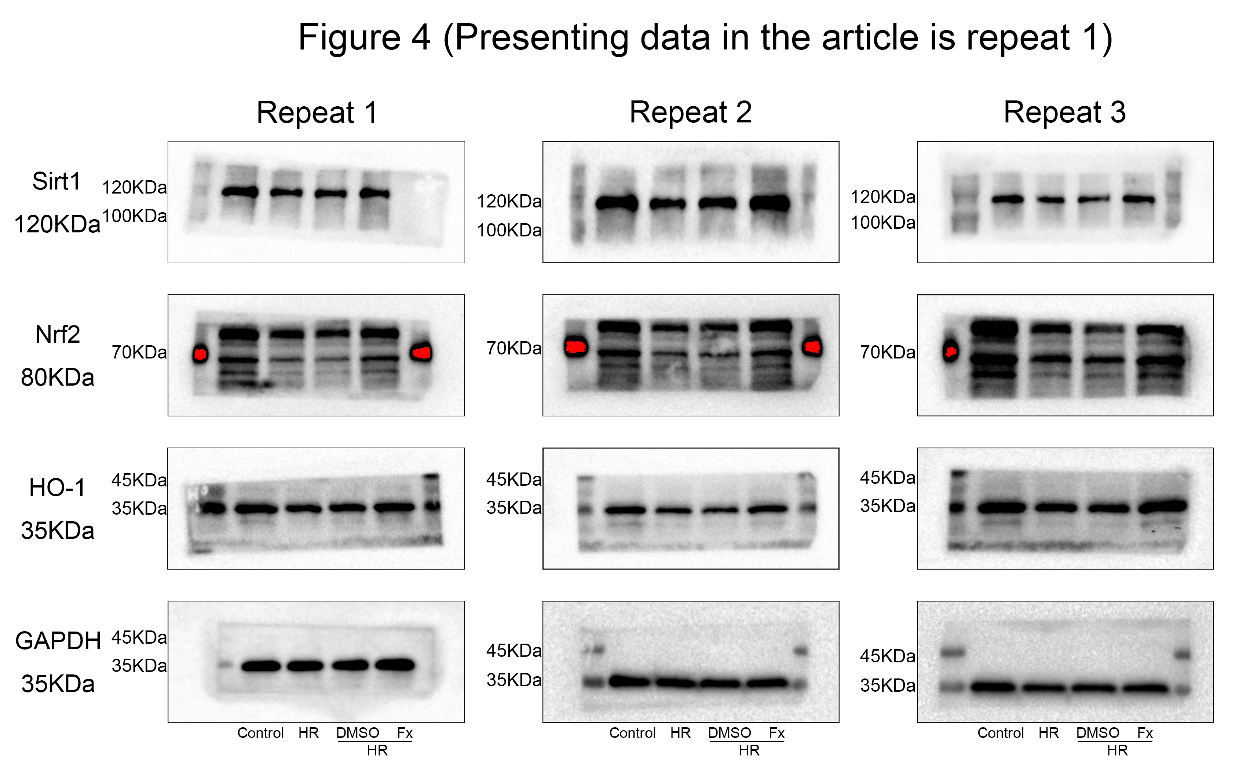


**Figure S5 Western blot data of Figure 5**
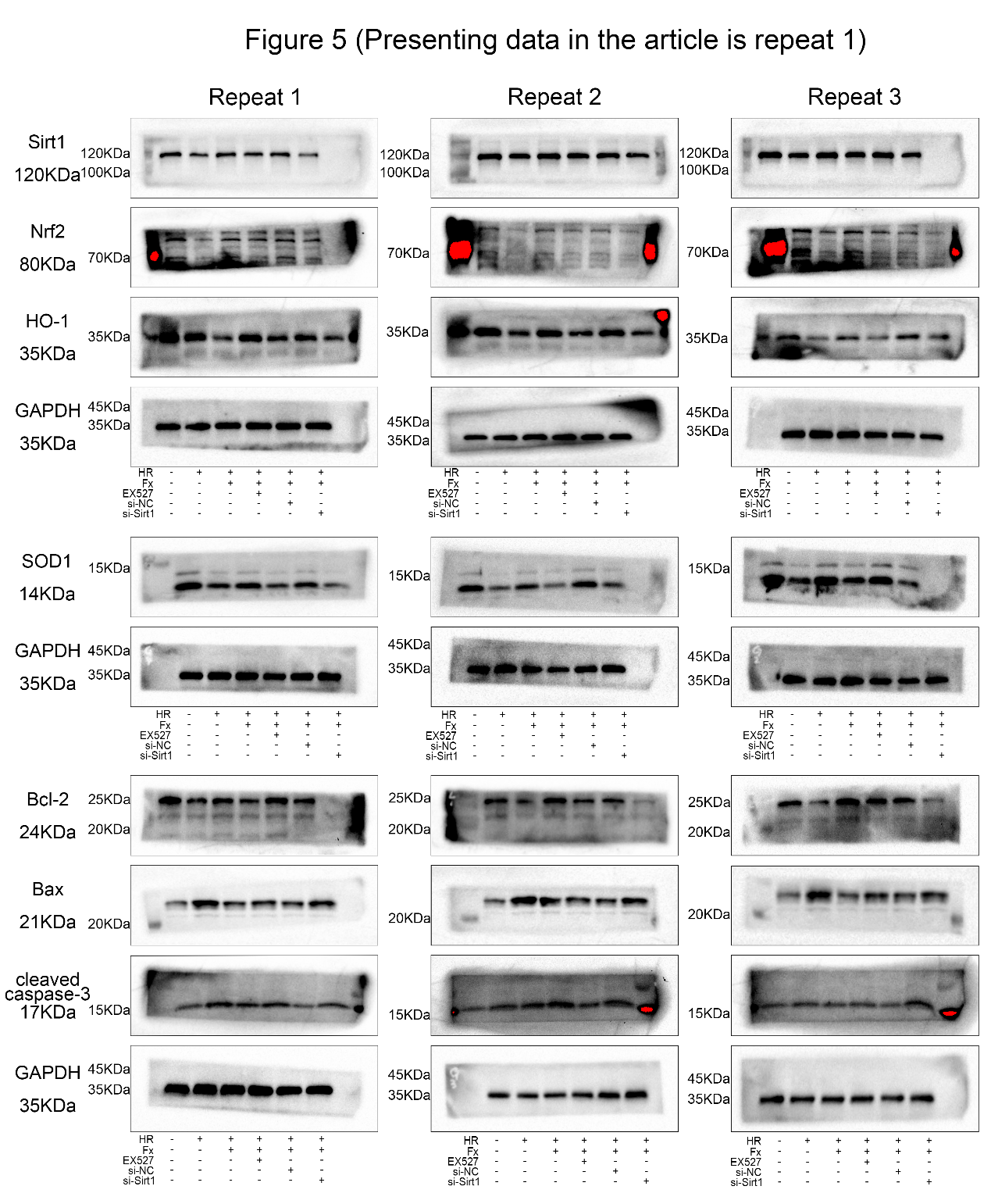


**Figure S6 Western blot data of Figure 6**
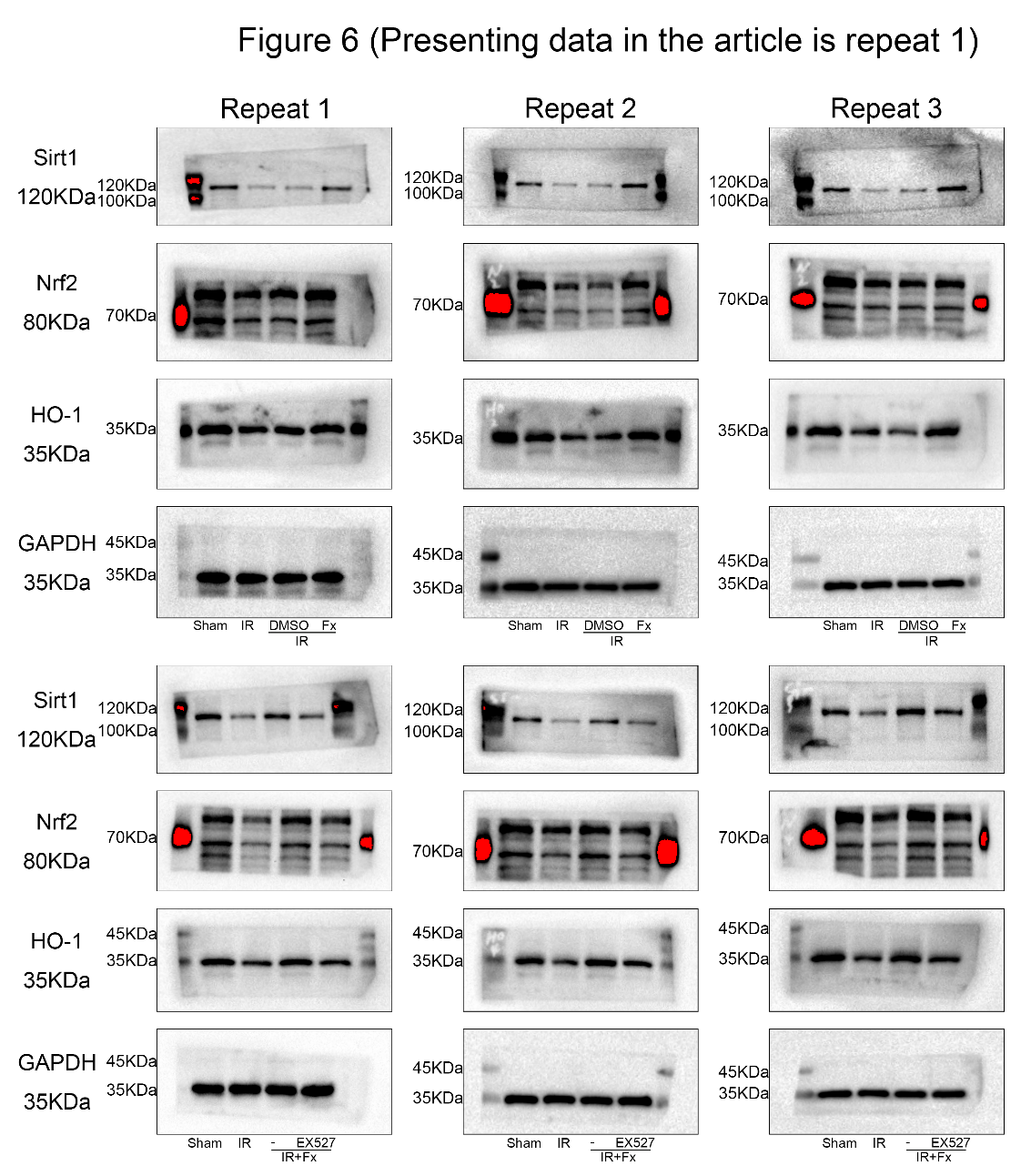


**Figure S7 Western blot data of Figure 7**
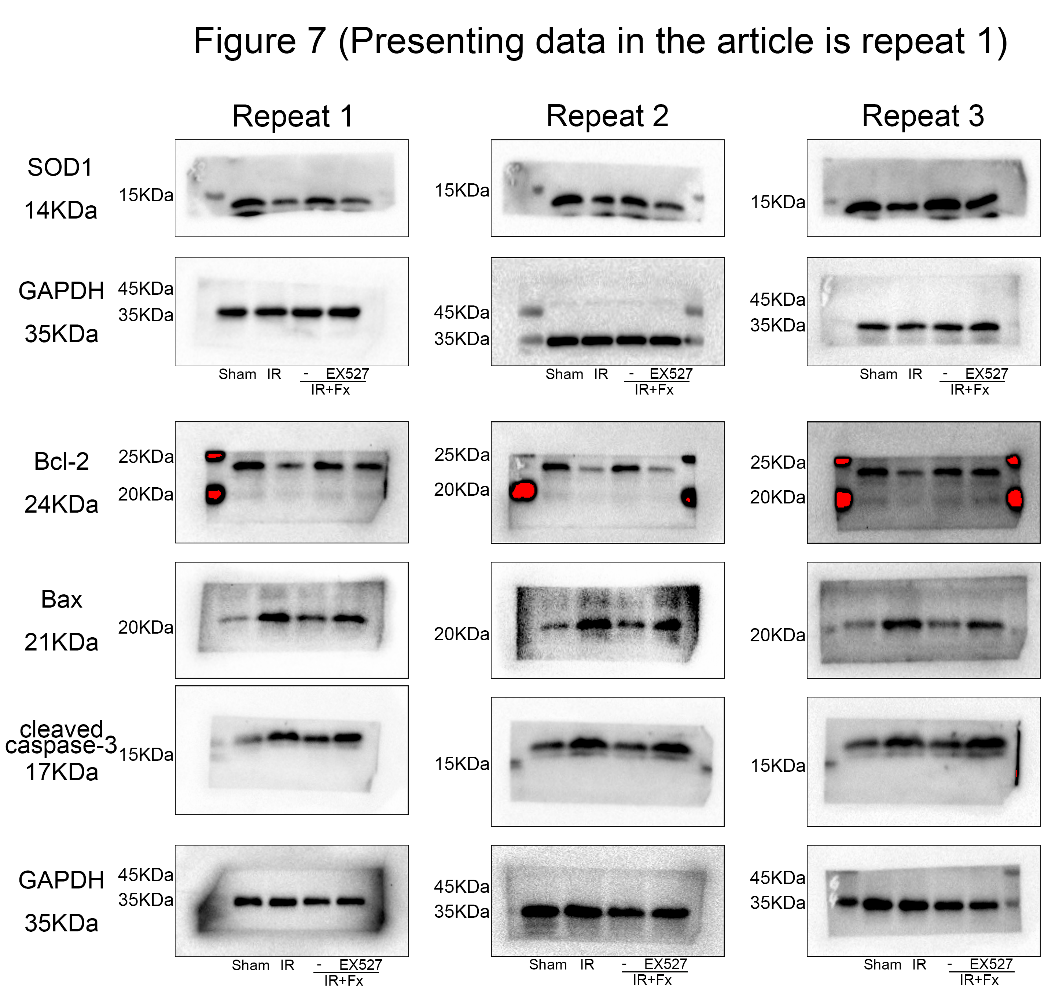

Supplement: Supplementary Materials — Figure S1: the chemical structure diagram of fucoxanthin. Figure S2: Western blot data of Figure 2. Figure S3: Western blot data of Figure 3. Figure S4: Western blot data of Figure 4. Figure S5: Western blot data of Figure 5. Figure S6: Western blot data of Figure 6. Figure S7: Western blot data of Figure 7. [file 7444430.f1.docx]
